# Endophyte Infection and Methyl Jasmonate Treatment Increased the Resistance of *Achnatherum sibiricum* to Insect Herbivores Independently

**DOI:** 10.3390/toxins11010007

**Published:** 2018-12-26

**Authors:** Junhua Qin, Man Wu, Hui Liu, Yubao Gao, Anzhi Ren

**Affiliations:** College of Life Sciences, Nankai University, Tianjin 300071, China; Junhua@mail.nankai.edu.cn (J.Q.); manwu@mail.nankai.edu.cn (M.W.); hui@mail.nankai.edu.cn (H.L.); ybgao@mail.nankai.edu.cn (Y.G.)

**Keywords:** *Achnatherum sibiricum*, methyl jasmonate, *Epichloë* endophyte, resistance, soluble sugar content, total phenolic

## Abstract

Alkaloids are usually thought to be responsible for protecting endophyte-infected (EI) grasses from their herbivores. For EI grasses that produce few alkaloids, can endophyte infection enhance their resistance to herbivores? Related studies are limited. In the Inner Mongolian steppe, *Achnatherum sibiricum* is highly infected by *Epichloë* endophytes, but produces few alkaloids. Locusts are the common insect herbivores of grasses. In this study, *A. sibiricum* was used as plant material. Methyl jasmonate (MJ, when applied exogenously, can induce responses similar to herbivore damage) treatment was performed. The effects of endophyte infection and MJ treatment on the resistance of *A. sibiricum* to *Locusta migratoria* were studied. We found that locusts preferred EF (endophyte-free) plants to EI plants in both choice and no-choice feeding experiments. Endophyte infection enhanced the resistance of *A. sibiricum* to locusts. Endophyte infection decreased soluble sugar concentrations, while it increased the total phenolic content and phenylalanine ammonia lyase (PAL) activity, which may contribute to the resistance of *A. sibiricum* to locusts. There was an interaction effect between MJ treatment and endophyte infection on the growth of the host. MJ treatment was a negative regulator of the plant growth-promoting effects of endophyte infection. There was no interaction effect between MJ treatment and endophyte infection on the defense characteristics of the host. In groups not exposed to locusts, MJ treatment and endophyte infection had a similar effect in decreasing the soluble sugar content, while increasing the total phenolic content and the PAL activity. In groups exposed to locusts, the effect of MJ treatment on the above characteristics disappeared, while the effect of endophyte infection became more obvious. All of these results suggest that even for endophytes producing few alkaloids, they could still increase the resistance of native grasses to insect herbivores. Furthermore, endophyte infection might mediate the defense responses of the host, independent of jasmonic acid (JA) pathways.

## 1. Introduction

*Epichloë* endophyte species are characterized by their endophytic lifestyles in aerial parts of cool-season grasses [1]. Up to 30% of cool-season grasses have been reported to be associated with *Epichloë* species [2]. In the symbionts, the grasses provide nutrients to the endophytes [3], and the endophytes can benefit the grasses by stimulating growth [4,5], and increasing resistance to abiotic and biotic stresses [6,7,8,9,10,11]. A remarkable characteristic of many *Epichloë* species is their ability to produce biologically active alkaloids, which contribute to the deterrence of herbivores [12,13,14]. Up to now, four classes of alkaloids have been found, including lolines, peramines, ergot alkaloids, and lolitrems [12,13,14,15].

Alkaloids are widely distributed in endophyte-infected grasses, but their profiles and concentrations vary considerably [16,17,18]. Siegel et al. surveyed 48 grass samples infected by different species of *Epichloë*, and they found that five symbionts had three classes of alkaloids, 19 contained two, 19 contained one, and five did not contain alkaloids [16]. Can endophyte infection enhance the herbivore deterrence of host plants that produce few alkaloids? The related studies are limited [19,20].

Jasmonates (jasmonic acid (JA) and methyl jasmonate (MJ)) are plant hormones synthesized naturally in response to biotic and abiotic stresses, and they influence plant growth and development [21,22,23,24]. The best-known function of jasmonates is their important role in herbivore-induced responses in plants [25,26,27,28]. When applied exogenously, jasmonates induce responses that are similar to those initiated following herbivore damage, and so provide a tool with which to induce defense-related responses in plants without the removal of lamina tissue [29,30,31,32,33]. One of the most important contributions of jasmonates is their stimulatory effect on plant secondary metabolites, including alkaloids [34,35,36]. However, there are few studies on the interactions between plant responses to jasmonates and to endophyte infection. The only relevant study was performed in tall fescue, *Lolium arundinaceum* [32], with a high concentrations of alkaloids [14], and this study found that endophyte-infected (EI) plants exposed to MJ were less resistant to aphids than unexposed plants, which indicated that endophyte-associated defense was compromised by jasmonates. Therefore, results from more endophyte-grass symbionts, producing few alkaloids, will expand upon the previous findings.

*Achnatherum sibiricum* (L.) Keng is a caespitose perennial grass that is widely distributed in the Inner Mongolia steppe of China. In our previous studies, we found that *A. sibiricum* is normally infected by two species of endophytes, *Epichloë gansuensis* and *Epichloë sibirica*, in its native populations [37,38]. As for the alkaloids in endophyte-infected *A. sibiricum*, the above-mentioned four classes of alkaloids in seeds and leaf sheaths have been analyzed, and no known alkaloids were detected in seeds or endophyte-infected plants when grown under normal conditions. After MJ treatment, only peramine was detected in one sample, and its concentration in the sheaths of infected plants was 0.4 mg/kg. It has been reported that the peramine alkaloid is a deterrent, and toxic to invertebrate herbivores only at levels greater than 3.0 mg/kg [17,18]. Therefore, the benefits of infection to the host related to herbivore deterrence via endophyte-related alkaloids seem improbable in *A. sibiricum*. In the native grasslands where *A. sibiricum* is distributed, locusts are common insect herbivores, and they often cause great damage to grasses, and compete with domestic animals for food resources. In the present study, endophyte-infected (EI) and endophyte-free (EF) *A. sibiricum* was used as the plant material. MJ treatment, including three levels (no MJ control, CK; low MJ concentration, MJL; and high MJ concentration, MJH), was performed. We addressed the following questions: (i) Does endophyte infection have a positive effect on the resistance of *A. sibiricum* to herbivores? (ii) Is there an interaction between MJ treatment and endophyte infection on the resistance of *A. sibiricum* to herbivores, and if so, is it antagonistic or synergistic?

## 2. Results

### 2.1. Leaf Consumption by L. migratoria

**Figure 1:**
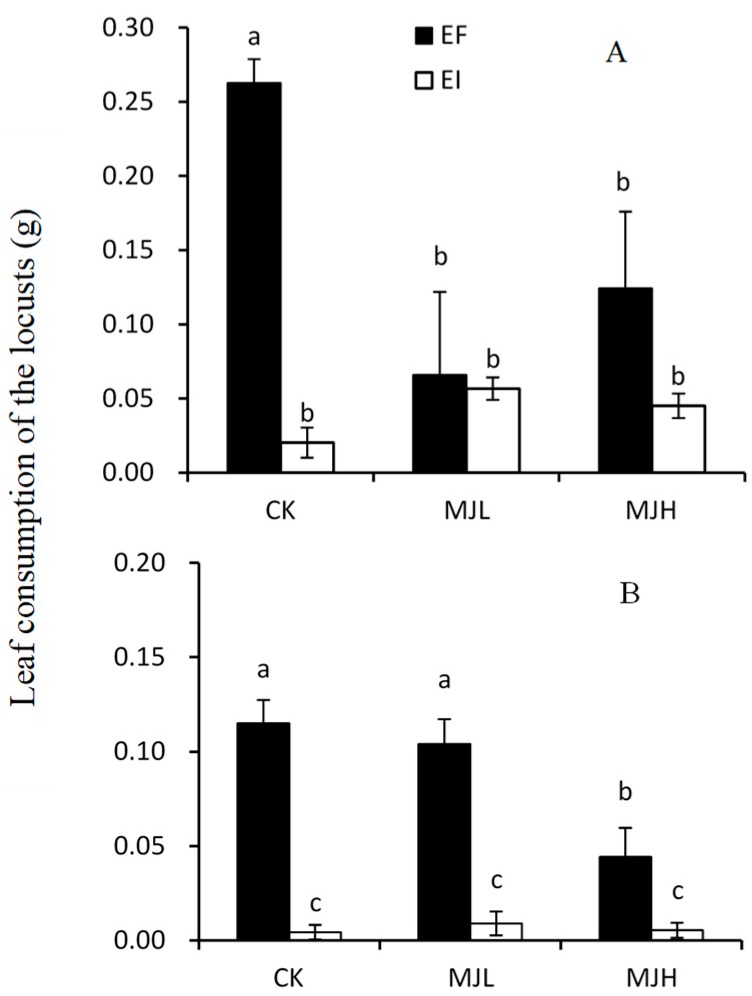
Comparison of the leaf consumption of endophyte-infected (EI) or endophyte-free (EF) *Achnatherum sibiricum* by the locusts, *L. migratoria*, in choice (**A**) and no-choice (**B**) tests under different MJ (methyl jasmonate) treatments (CK, no MJ control; MJL, low MJ concentration; MJH, high MJ concentration). Different lower-case letters denote means that are significantly different (*p* < 0.05).

In the choice feeding experiment, the amount of mass consumed by *L*. *migratoria* was significantly affected by endophyte infection (*F* = 17.405, *p* < 0.01), as well as the interaction of the endophyte and MJ treatment (*F* = 6.841, *p* = 0.010). The highest rate of consumption occurred in EF plants under the control treatment. Both endophyte infection and MJ treatment significantly reduced the palatability of *A. sibiricum*. There was no difference in leaf consumption between the EI plants and the EF plants that were sprayed with MJ, as shown in Figure 1A.

In the no-choice feeding experiment, the amount of mass consumed by *L*. *migratoria* was significantly affected by endophyte infection (*F* = 117.35, *p* < 0.01), MJ treatment (*F* = 8.688, *p* < 0.01), and the interaction between endophyte infection and MJ treatment (*F* = 8.348, *p* < 0.01). Endophyte infection significantly reduced the leaf consumption of the host plants, and this effect was not affected by MJ treatment. For EF plants, the amount of biomass consumed by the locusts decreased with MJ treatment (*F* = 9.560, *p* < 0.01), but a significant reduction occurred only in the MJH treatment, as shown in Figure 1B.

### 2.2. Growth and Biomass

**Figure 2:**
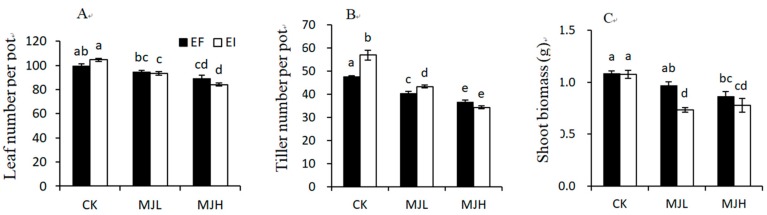
Comparison of leaf number (**A**), tiller number (**B**), and shoot biomass (**C**) of endophyte-free (EF) and endophyte-infected (EI) *Achnatherum sibiricum* under different MJ (methyl jasmonate) treatments (CK, no MJ control; MJL, low MJ concentration; MJH, high MJ concentration). Different lower-case letters denote means that are significantly different (*p* < 0.05).

Plant height was only significantly affected by MJ treatment, as shown in Table 1, and MJ treatment reduced plant height (CK = 31.16 cm; MJL = 27.98 cm; MJH = 25.64 cm). Leaf number, tiller number, and shoot biomass were significantly affected by the interaction between MJ treatment and endophyte infection, as shown in Table 1. MJ treatment inhibited the vegetative growth of both the EI and EF plants, but the inhibition degree was stronger for the EI than the EF plants. MJL significantly reduced the leaf number and shoot biomass of the EI plants, but a significant reduction in the leaf number and shoot biomass of the EF plants occurred only with the MJH treatment. MJL and MJH resulted in a 15% and 23% reduction in tiller number for the EF plants, respectively, and a 24% and 40% reduction in tiller number for the EI plants, respectively, as shown in Figure 2.

### 2.3. Physiological Variables

**Figure 3:**
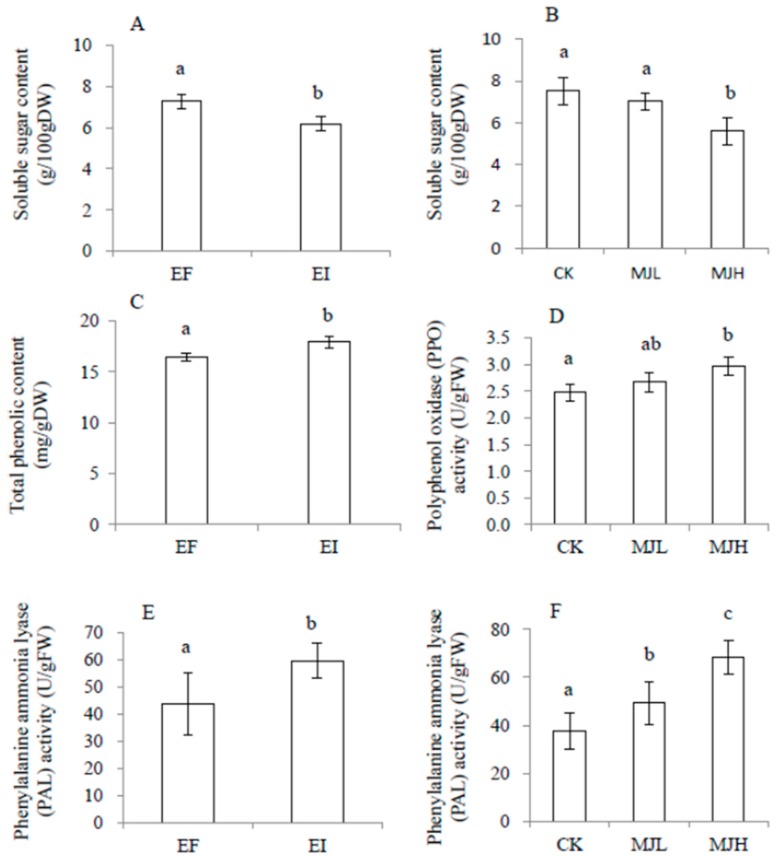
Comparison of soluble sugar content (**A**,**B**), total phenolic content (**C**), polyphenol oxidase (PPO, **D**), and phenylalanine ammonia lyase (PAL, **E**,**F**) of *Achnatherum sibiricum* under different endophyte statuses (EI, endophyte-infected; EF, endophyte-free) or MJ (methyl jasmonate) treatments (CK, no MJ control; MJL, low MJ concentration; MJH, high MJ concentration) without locust feeding. Different lower-case letters denote means that are significantly different (*p* < 0.05).

**Figure 4:**
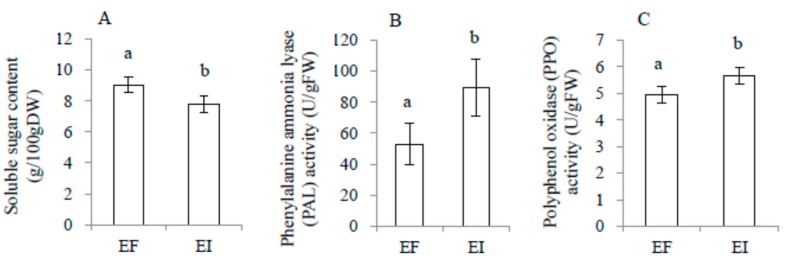
Comparison of soluble sugar content (**A**), phenylalanine ammonia lyase (PAL, **B**), and polyphenol oxidase (PPO, **C**) of endophyte-infected (EI) or endophyte-free (EF) *Achnatherum sibiricum* with locust feeding. Different lower-case letters denote means that are significantly different (*p* < 0.05).

There was no significant interaction effect between endophyte infection and MJ treatment on soluble sugar, total phenolics, phenylalanine ammonia lyase (PAL), and polyphenol oxidase (PPO), as shown in Table 2. In the no-feeding groups, soluble sugar content was significantly reduced by both endophyte infection and MJ treatment, while PAL was significantly enhanced by both endophyte infection and MJ treatment. Total phenolics and PPO were enhanced by endophyte infection and MJ treatment, separately, as shown in Figure 3. After locust feeding, the above physiological variables were significantly affected only by endophyte infection, but not by MJ treatment. Furthermore, endophyte infection reduced the plants’ soluble sugar content, while it enhanced PPO and PAL activities, as shown in Figure 4.

## 3. Discussion

*Epichloë* endophytes can protect the host grass from insect herbivores, and endophyte-associated alkaloids are thought to be primarily responsible for feeding deterrence [14,39,40,41,42,43,44]. In our experiments, only peramine was detected in one sample, and its concentration is not high enough to deter insects [17,18]. However, we found that infected *A. sibiricum* had significant resistance to locusts in both the choice and no-choice tests, as shown in Figure 1, which suggests that aside from alkaloid defense, additional mechanisms are likely to be involved in endophyte-associated insect deterrence.

Phytophagous insects depend on plants for their nutrients, so that their growth and development are affected directly by the makeup of the food source. Primary metabolites such as carbohydrates are important nutrients for animal growth [45]. Secondary metabolites such as plant phenols play an important role in the resistance of the host plant to herbivores [46,47]. In the present study, we found that endophyte infection significantly increased the total phenolic content in the host plants, as shown in Table 2 and Figure 3. Similar results have been reported in perennial ryegrass, *Lolium perenne* [48,49] and tall fescue [50]. Moreover, PAL and PPO are enzymes that are involved in phenol oxidation, and they are correlated with plant defense mechanisms [51]. Higher levels of PAL [52,53] and PPO activity [54,55,56] have been reported to be associated with the resistance of plants to insects. Here, the higher phenol concentration, PAL and PPO activity, combined with the lower soluble sugar concentration of the EI leaves may be responsible for the higher locust deterrence of *A. sibiricum*.

JA is a ubiquitous wound hormone. Repeated wounding of mature leaves induces a rapid increase in endogenous JA concentrations, thus resulting in reduced leaf growth [57]. Exogenous applications of JA or MJ can mimic the temporal and quantitative characteristics of endogenous JA responses [58,59,60,61]. In the present study, MJ treatment inhibited the vegetative growth of both the EI and EF plants, as shown in Table 1 and Figure 2. This is in agreement with other studies that found a reduction in both root growth [62,63] and shoot growth [64,65,66] under exogenous JA treatments. On the contrary, jasmonates might act as negative regulators of plant primary metabolic products, such as sugars [23,67,68,69,70,71] and as positive regulators of plant secondary metabolic products, such as phenolic compounds [72]. In the present study, we also found that MJ treatment decreased soluble sugar content, while it increased the PPO and PAL activities of the treated plants, as shown in Table 2 and Figure 3.

A JA-dependent pathway is mainly induced by chewing insects [73,74,75]. Additionally, these hormones also regulate the interactions of plants with beneficial organisms, such as the symbiosis with arbuscular mycorrhizal fungi (AMF) [76,77] and root endophytes [78]. For example, mycorrhization had a positive effect on floral traits, but its benefits were lost with JA application. JA signaling and JA biosynthesis mutants caused a significant reduction in *Piriformospora indica* colonization [78]. In the present study, MJ treatment reduced the growth-promoting effects of endophyte infection. Similar results have also been found regarding the effects of the root endophyte *P. indica* on *Nicotiana attenuata* [79]. Recently, *P. indica* has been found to promote the growth of JA-insensitive mutants more strongly than wild-type (WT) rice [80]. All these results suggest that JA signaling is a negative regulator of the plant growth-promoting effects induced by endophytes. It has been suggested that auxin and gibberellic acid (GA) released by endophytic fungi may explain growth enhancements in host plants [81,82,83]. The negative effect of JA on the growth-promoting effects of endophyte infection might be mediated via antagonistic interactions with the auxin/GA signaling pathway [84,85,86].

In the present study, MJ treatment and endophyte infection had a similar effect in decreasing plants’ soluble sugar contents, while increasing the total phenolic content and PAL activity in the no-feeding groups, but there were no interactions between MJ treatment and endophyte infection, as shown in Table 2 and Figure 3. In a previous study, JA inhibitors were found to suppress both JA and volatile oil production, but fungal inoculation could still induce volatile oils [87]. All of these results suggest that endophyte infection might mediate the defense responses of the host via different pathways, such as H_2_O_2_-dependent pathways [87]. In the feeding groups, the effects of MJ treatment on the above characteristics disappeared, while the effects of endophyte infection became more obvious. The reason for this might be that endophyte pre-infection and late locust feeding led to a much stronger induction of the plants’ defense responses, as similar results have also been found in AMF-plant systems [77]. Certainly, interactions among endophytes, endogenous hormone levels, and the host defense traits depend on the specific species, strain, or very likely, the plant-endophyte combination, and further research using more combinations of plants and endophytes is still needed.

## 4. Conclusions

In this study, we found that endophytes significantly improved the resistance of the host to locusts, although the host produced few alkaloids. Here, the lower soluble sugar concentration, higher total phenolics concentration, and higher PAL activity contributed to the higher resistance of the host plants to the locusts. MJ treatment inhibited the vegetative growth of both the EI and EF plants. MJ treatment is a negative regulator of the plant growth-promoting effects of endophyte infection. However, regarding plants’ defense characteristics, the effects of endophyte infection may be independent of JA pathways.

## 5. Materials and Methods

### 5.1. Plant Material and Treatment

Seeds of *A. sibiricum* were collected from the National Hulunber Grassland Ecosystem Observation and Research Station of China. After examining the endophyte status of both seeds and seedlings following staining with aniline blue [88], we found that the endophyte infection rate was 100% (infection rate = the number of infected seedlings (or seeds)/total seedlings (or seeds) [88]). Endophyte-free (EF) seeds were obtained by high temperature treatment [89]. The seeds were planted in white plastic pots (20 cm in diameter and 20 cm in depth) filled with vermiculite. After 30 days’ cultivation, the endophyte infection status of each plant was further inspected, and 10 plants of approximately equal size were maintained in each pot. Meantime, MJ treatments were processed. The experiment had a completely randomized 2 × 3 × 2 factorial design, with endophyte infection status (EI and EF), MJ treatment (no MJ control, CK; low MJ concentration, MJL; and high MJ concentration, MJH), and locust feeding (no-feeding and feeding) as the variables. There were six replicates per treatment group for the choice feeding experiment, and five replicates for the no-choice feeding experiment. The experimental plants were watered and fertilized with modified Hoagland nutrient solution as needed. The composition of the nutrient solution was (μM) [90]: Ca(NO_3_)_2_ 5000, KNO_3_ 5000, MgSO4·7H_2_O 2500, KH_2_PO_4_ 2000, Na_2_C_10_H_l4_O_3_N_2_ 29, FeSO_4_·7H_2_O 20, H_3_BO_3_ 45, MnSO_4_ 6.6, ZnSO_4_·7H_2_O 0.8, H_2_MoO_4_ 0.6, CuSO_4_·5H_2_O 0.4. The experiment was conducted in a greenhouse at Nankai University, Tianjin, China, with temperatures ranging from 19 to 25 °C, a relative humidity of 40–50%, and natural daylight.

### 5.2. Locusta Migratoria

Locust eggs were purchased from a local pet shop. Fourth instar nymphs were used in the experiments.

### 5.3. Methyl Jasmonate (MJ) Treatment

MJ (purity: 98% HPLC-grade) was bought from 3B Pharmachem (Wuhan, China) International Co., Ltd. MJ was firstly dissolved in absolute ethyl alcohol, and then dispersed in water to the desired concentration. MJ was applied with a backpack sprayer to plants until run-off; if necessary, the surrounding leaflets were shielded with a piece of paper. Control plants were sprayed with water that contained alcohol at the same concentration used for the MJ treatments. Neighboring plants were shielded from the spray with a large sheet of plastic. Plants were divided randomly into three treatment groups: control (no MJ), low MJ (25 mg/L, 0.025 mg of MJ per plant), and high MJ (400 mg/L, 0.4 mg of MJ per plant).

### 5.4. Choice Feeding Experiment

After starvation for 24 h, the fourth-instar locust nymphs were transferred to transparent plastic containers (19 cm height and 8 cm diameter). Locust nymphs were provided with 0.5 g leaf blades per pot per treatment as food. These leaf blades were cut 24 h after MJ treatment, and were transferred to the above containers simultaneously with the locusts. There were 12 plastic containers in total. Eight locusts were added to each of the six containers. Another six containers were used as a control to calculate the reduced leaf quality due to evaporation. After 1 h of exposure to herbivory, plants were harvested to record the fresh biomass.

### 5.5. No-Choice Feeding Experiment

Twenty-four hours after MJ treatment, we equipped every pot with a steel frame (45 cm height and 20 cm diameter), which was covered with a nylon stocking. Three fourth-instar locust nymphs, starved for 24 h, were introduced into half of these resulting cages. The feeding exposure lasted 3 h. Before and after the feeding, we immediately measured the length (L) and maximum width (W) of each leaf with a ruler. We estimated leaf area (S) according to the following equation: S = K (L × W), where K is 0.9, based on our previous study using *A. sibiricum* plants of similar sizes (unpublished data). At the same time, 10 fully expanded leaves per no-feeding pot were chosen to measure the area and were weighed separately for determination of specific leaf area (SLA). Leaf mass consumed was obtained from consumed leaf area divided by SLA.

### 5.6. Growth and Biomass

Growth variables were measured only in the no-feeding plants in the no-choice experiment. Twenty days after MJ treatment, regular measurement of tiller number, leaf number, and shoot height of the longest tiller were made on all ramets. Subsequently, the shoot and the root were harvested separately. The harvested material was oven-dried at 80 °C for biomass measurement [91].

### 5.7. Physiological Variables

Physiological variables were sampled before and 24 h after no-choice feeding, separately. The soluble sugar concentration was analyzed using the colorimetric method [92] [93]. The total phenolic concentration was determined according to Malinowski et al. [50]. Polyphenol oxidase (PPO) activities were determined spectrophotometrically according to the methods described in Thaler et al. [94], and phenylalanine ammonia lyase (PAL) was determined using modified procedures described by Assis et al. [95].

### 5.8. Statistical Analysis

All statistical analyses were performed with SPSS software (Version 21.0, SPSS, Chicago, IL, USA, 2012). Endophyte status and MJ treatment were analyzed using two-way analysis of variance (ANOVA). The differences between the means among different factors were compared using Duncan’s multiple-range tests, and significance was set at *p* < 0.05.

## Figures and Tables

**Table 1:** Two-way ANOVA (analysis of variance) for the vegetative growth of endophyte-infected (EI) or endophyte-free (EF) *Achnatherum sibiricum* under MJ treatments.

Treatment	Plant Height		Leaf Number		Tiller Number		Shoot Biomass		Root Biomass	
	*F*	*p*	*F*	*p*	*F*	*p*	*F*	*p*	*F*	*p*
Endophyte (E)	2.155	0.155	0.070	0.793	22.617	**<0.01**	11.170	**<0.01**	17.078	**<0.01**
MJ	12.007	**<0.01**	35.715	**<0.01**	181.574	**<0.01**	24.986	**<0.01**	0.350	0.708
E × MJ	1.199	0.319	3.449	**0.048**	19.904	**<0.01**	3.832	**0.036**	3.145	0.061

MJ denotes methyl jasmonate. *F* and *p* denote *F*-ratio and *p*-value produced in the ANOVA test, respectively. Significant *p*-values are in bold.

**Table 2:** Two-way ANOVA for the physiological responses of endophyte-infected (EI) or endophyte-free (EF) *Achnatherum sibiricum* under MJ treatments.

Treatment	Soluble Sugar		Total Phenolics		Phenylalanine Ammonia Lyase (PAL)		Polyphenol Oxidase (PPO)	
	*F*	*p*	*F*	*p*	*F*	*p*	*F*	*p*
No feeding								
Endophyte (E)	6.631	**0.017**	5.656	**0.026**	7.841	**0.012**	3.797	0.063
MJ	7.212	**0.004**	2.908	0.075	10.215	**0.001**	5.012	**0.015**
E × MJ	0.111	0.896	0.964	0.396	2.790	0.088	0.671	0.520
Feeding								
E	8.529	**0.008**	2.555	0.124	10.780	**0.003**	6.589	**0.019**
MJ	0.572	0.573	0.215	0.808	1.377	0.272	1.735	0.205
E × MJ	0.651	0.531	0.466	0.634	2.043	0.152	0.347	0.711

MJ denotes methyl jasmonate. *F* and *p* denote *F*-ratio and *p*-value produced in the ANOVA test, respectively. Significant *p*-values are in bold.

## References

[1] Carroll G. (1988). Fungal endophytes in stems and leaves: From latent pathogen to mutualistic symbiont. Ecology.

[2] Leuchtmann A. (1992). Systematics, distribution, and host specificity of grass endophytes. Nat. Toxins.

[3] Schardl C.L., Leuchtmann A., Spiering M.J. (2004). Symbioses of grasses with seedborne fungal endophytes. Annu. Rev. Plant Biol..

[4] Saikkonen K., Wali P., Helander M., Faeth S.H. (2004). Evolution of endophyte-plant symbioses. Trends Plant Sci..

[5] Clay K. (1990). Fungal endophytes of grasses. Annu. Rev. Ecol. Syst..

[6] Malinowski D.P., Belesky D.P. (1999). Endophyte infection enhances the ability of tall fescue to untilize sparingly available phosphorus. J. Plant Nutr..

[7] Hesse U., Schöberlein W., Wittenmayer L., Förster K., Warnstorff K., Diepenbrock W., Merbach W. (2003). Effects of *Neotyphodium* endophytes on growth, reproduction and drought-stress tolerance of three *Lolium perenne* L. genotypes. Grass Forage Sci..

[8] Burns J.C., Fisher D.S. (2006). Intake and digestion of ‘Jesup’ tall fescue hays with a novel fungal endophyte, without an endophyte, or with a wild-type endophyte. Crop. Sci..

[9] Gibert A., Hazard L. (2011). Endophyte infection of *Festuca eskia* enhances seedling survival to drought and cutting at the expense of clonal expansion. J. Plant Ecol..

[10] Worchel E.R., Giauque H.E., Kivlin S.N. (2013). Fungal symbionts alter plant drought response. Microb. Ecol..

[11] Rúa M.A., Mcculley R.L., Mitchell C.E. (2013). Fungal endophyte infection and host genetic background jointly modulate host response to an aphid-transmitted viral pathogen. J. Ecol..

[12] Siegel M.R., Latch G.C.M., Bush L.P., Fannin F.F., Rowan D.D., Tapper B.A., Bacon C.W., Johnson M.C. (1990). Fungal endophyte-infected grasses: Alkaloid accumulation and aphid response. J. Chem. Ecol..

[13] Bush L.P., Wilkinson H.H., Schardl C.L. (1997). Bioprotective alkaloids of grass-fungal endophyte symbioses. Plant Physiol..

[14] Clay K., Schardl C. (2002). Evolutionary origins and ecological consequences of endophyte symbiosis with grasses. Am. Nat..

[15] Rowan D.D. (1993). Lolitrems, peramine and paxilline: Mycotoxins of the ryegrass/endophyte interaction. Agric. Ecosyst. Environ..

[16] Siegel M.R., Bush L.P., Carroll G.C. (1997). Toxin production in grass/endophyte associations. Plant Relationships. The Mycota (A Comprehensive Treatise on Fungi as Experimental Systems for Basic and Applied Research).

[17] Siegel M.R., Bush L.P., Romeo J.T. (1996). Defensive chemicals in grass-fungal endophyte associations. Phytochemical Diversity and Redundancy in Ecological Interactions. Recent Advances in Phytochemistry.

[18] Rowan D.D., Latch G.C.M. (1994). Utilization of endophyte-infected perennial ryegrass for increased insect resistance. Biotechnology of Endophytic Fungi of Grasses.

[19] Faeth S.H., Shochat E. (2010). Inherited microbial symbionts increase herbivore abundances and alter arthropod diversity on a native grass. Ecology.

[20] Li T., Blande J.D., Gundel P.E., Helander M., Saikkonen K. (2014). *Epichloë* endophytes alter inducible indirect defences in host grasses. PLoS ONE.

[21] Pozo M.J., Loon L.C.V., Pieterse C.M.J. (2004). Jasmonates-signals in plant-microbe interactions. J. Plant Growth Regul..

[22] Kiers T.E., Adler L.S., Grman E.L., Van Der Heijden M.G.A. (2010). Manipulating the jasmonate response: How do methyl jasmonate additions mediate characteristics of aboveground and belowground mutualisms?. Funct. Ecol..

[23] Machado R.A.R., Arce C.C.M., Ferrieri A.P., Baldwin I.T., Matthias E. (2015). Jasmonate-dependent depletion of soluble sugars compromises plant resistance to *Manduca sexta*. New Phytol..

[24] Balbi V., Devoto A. (2008). Jasmonate signalling network in Arabidopsis thaliana: Crucial regulatory nodes and new physiological scenarios. New Phytol..

[25] Halitschke R., Baldwin I.T. (2004). Jasmonates and related compounds in plant-insect interactions. J. Plant Growth Regul..

[26] Wu J., Baldwin I.T. (2010). New insights into plant responses to the attack from insect herbivores. Annu. Rev. Genet..

[27] Ballaré C.L. (2011). Jasmonate-induced defenses: A tale of intelligence, collaborators and rascals. Trends Plant. Sci..

[28] Li S.J., Xue X., Ren S.X., Cuthbertson A.G.S., Dam N.M.V., Qiu B.L. (2013). Root and shoot jasmonic acid induced plants differently affect the performance of *Bemisia tabaci* and its parasitoid *Encarsia formosa*. Basic Appl. Ecol..

[29] Mcconn M., Creelman R.A., Bell E., Mullet J.E., Browse J. (1997). Jasmonate is essential for insect defense in Arabidopsis. Proc. Natl. Acad. Sci. USA.

[30] Li L., Li C., Lee G.I., Howe G.A. (2002). Distinct roles for jasmonate synthesis and action in the systemic wound response of tomato. Proc. Natl. Acad. Sci. USA.

[31] Qiu B.L., Jeffreya H., Ciskae R., Louiseem V., Nicolem V.D. (2009). Nonlinear effects of plant root and shoot jasmonic acid application on the performance of *Pieris brassicae* and its parasitoid *Cotesia glomerata*. Funct. Ecol..

[32] Simons L., Bultman T.L., Sullivan T.J. (2008). Effects of methyl Jasmonate and an endophytic fungus on plant resistance to insect herbivores. J. Chem. Ecol..

[33] Moore J.P., Paul N.D., Whittaker J.B., Taylor J.E. (2003). Exogenous jasmonic acid mimics herbivore-induced systemic increase in cell wall bound peroxidase activity and reduction in leaf expansion. Funct. Ecol..

[34] Chen H., Jones A.D., Howe G.A. (2006). Constitutive activation of the jasmonate signaling pathway enhances the production of secondary metabolites in tomato. FEBS Lett..

[35] Paschold A., Halitschke R., Baldwin I.T. (2007). Co(i)-ordinating defenses: NaCOI1 mediates herbivore- induced resistance in Nicotiana attenuata and reveals the role of herbivore movement in avoiding defenses. Plant J..

[36] Zhang H., Hedhili S., Montiel G., Zhang Y., Chatel G., Pré M., Gantet P., Memelink J. (2011). The basic helix-loop-helix transcription factor CrMYC2 controls the jasmonate-responsive expression of the *ORCA* genes that regulate alkaloid biosynthesis in *Catharanthus roseus*. Plant. J..

[37] Zhang X., Ren A.Z., Wei Y.K., Lin F., Li C., Liu Z.J., Gao Y.B. (2009). Taxonomy, diversity and origins of symbiotic endophytes of *Achnatherum sibiricum* in the Inner Mongolia Steppe of China. FEMS Microbiol. Lett..

[38] Li X., Zhou Y., Zhu M., Qin J., Ren A., Gao Y. (2015). Stroma-bearing endophyte and its potential horizontal transmission ability in *Achnatherum sibiricum*. Mycologia.

[39] Sullivan T.J., Rodstrom J., Vandop J., Librizzi J., Graham C., Schardl C.L., Bultman T.L. (2007). Symbiont-mediated changes in *Lolium arundinaceum* inducible defenses: Evidence from changes in gene expression and leaf composition. New Phytol..

[40] Schardl C.L., Young C.A., Faulkner J.R., Florea S., Pan J. (2012). Chemotypic diversity of epichloae, fungal symbionts of grasses. Fungal Ecol..

[41] Saikkonen K., Gundel P.E., Helander M. (2013). Chemical ecology mediated by fungal endophytes in grasses. J. Chem. Ecol..

[42] Wilkinson H.H., Siegel M.R., Blankenship J.D., Mallory A.C., Bush L.P., Schardl C.L. (2000). Contribution of fungal loline alkaloids to protection from aphids in a grass-endophyte mutualism. Mol. Plant Microbe Interact..

[43] Prestidge R.A., Gallagher R.T. (1988). Endophyte fungus confers resistance to ryegrass: Argentine stem weevil larval studies. Ecol. Entomol..

[44] Shymanovich T., Saari S., Lovin M.E., Jarmusch A.K., Jarmusch S.A., Musso A.M., Charlton N.D., Young C.A., Cech N.B., Faeth S.H. (2015). Alkaloid variation among epichloid endophytes of sleepygrass (*Achnatherum robustum*) and consequences for resistance to insect herbivores. J. Chem. Ecol..

[45] Roeder K.A., Behmer S.T. (2014). Lifetime consequences of food protein-carbohydrate content for an insect herbivore. Funct. Ecol..

[46] Ballare C.L., Scopel A.L., Stapleton A.E., Yanovsky M.J. (1996). Solar ultraviolet-B radiation affects seedling emergence, DNA integrity, plant morphology, growth rate, and attractiveness to herbivore insects in *Datura ferox*. Plant. Physiol..

[47] Izaguirre M.M., Mazza C.A., Svatos A., Baldwin I.T., Ballaré C.L. (2007). Solar ultraviolet-B radiation and insect herbivory trigger partially overlapping phenolic responses in *Nicotiana attenuate* and *Nicotiana longiflora*. Ann. Bot..

[48] Rasmussen S., Parsons A.J., Fraser K., Xue H., Newman J.A. (2008). Metabolic profiles of *Lolium perenne* are differentially affected by nitrogen supply, carbohydrate content, and fungal endophyte infection. Plant Physiol..

[49] Pańka D., Piesik D., Jeske M., Baturo-Cieśniewska A. (2013). Production of phenolics and the emission of volatile organic compounds by perennial ryegrass (*Lolium perenne* L.)/*Neotyphodium lolii* association as a response to infection by *Fusarium poae*. J. Plant Physiol..

[50] Malinowski D.P., Alloush G.A., Belesky D.P. (1998). Evidence for chemical changes on the root surface of tall fescue in response to infection with the fungal endophyte *Neotyphodium coenophialum*. Plant Soil.

[51] Karban R., Kuc J., Agrawal A.A. (1999). Induced resistance against pathogens and herbivores: An overview. Induced Plant Defenses Against Pathogens and Herbivores.

[52] Mercedes E.C., Sylvia V.C., Victor H.A. (2003). Relationships between aalicylic acid content, phenylalanine ammonia-lyase (PAL) activity, and resistance of barley to aphid infestation. J. Agric. Food Chem..

[53] Tang Y., Zou J., Zhang L., Li Z., Ma C., Ma N. (2012). Anti-fungi activities of *Bacillus thuringiensis* H_3_ chitinase and immobilized chitinase particles and their effects to rice seedling defensive enzymes. J. Nanosci. Nanotechnol..

[54] Haruta M., Pedersen J.A., Constabel C.P. (2001). Polyphenol oxidase and herbivore defense in trembling aspen (*Populus tremuloides*): CDNA cloning, expression, and potential substrates. Physiol. Plant.

[55] Zhang S.Z., Fan Z., Hua B.Z. (2008). Enhancement of phenylalanine ammonia lyase, polyphenoloxidase, and peroxidase in cucumber seedlings by *Bemisia tabaci* (Gennadius) (Hemiptera: Aleyrodidae) infestation. J. Integr. Agric..

[56] Constabel C.P., Yip L., Patton J.J., Christopher M.E. (2000). Polyphenol oxidase from hybrid poplar. Cloning and expression in response to wounding and herbivory. Plant Physiol..

[57] Zhang Y., Turner J.G. (2008). Wound-induced endogenous jasmonates stunt plant growth by inhibiting mitosis. PLoS ONE.

[58] Bruinsma M., Posthumus M.A., Mumm R., Mueller M.J., Loon J.J.A.V., Dicke M. (2009). Jasmonic acid-induced volatiles of *Brassica oleracea* attract parasitoids: Effects of time and dose, and comparison with induction by herbivores. J. Exp. Bot..

[59] Matsuura H., Aoi A., Satou C., Nakaya M., Masuta C., Nabeta K. (2009). Simultaneous UPLC MS/MS analysis of endogenous jasmonic acid, salicylic acid, and their related compounds. Plant. Growth Regul..

[60] Henkes G.J., Thorpe M.R., Minchin P.E., Schurr U., Roese U.S. (2008). Jasmonic acid treatment to part of the root system is consistent with simulated leaf herbivory, diverting recently assimilated carbon towards untreated roots within an hour. Plant Cell Environ..

[61] Zhang Z.P., Baldwin I.T. (1997). Transport of [2- ^14^C]jasmonic acid from leaves to roots mimics wound-induced changes in endogenous jasmonic acid pools in *Nicotiana sylvestris*. Planta.

[62] Staswick P.E., Su W., Howell S.H. (1992). Methyl jasmonate inhibition of root growth and induction of a leaf protein are decreased in an *Arabidopsis thaliana* mutant. Proc. Natl. Acad. Sci. USA.

[63] Uppalapati S.R., Ayoubi P., Weng H., Palmer D.A., Mitchell R.E., Jones W., Bender C.L. (2005). The phytotoxin coronatine and methyl jasmonate impact multiple phytohormone pathways in tomato. Plant J..

[64] van Kleunen M., Ramponi G., Schmid B. (2004). Effects of herbivory simulated by clipping and jasmonic acid on *Solidago canadensis*. Basic Appl. Ecol..

[65] Cho K., Agrawal G.K., Shibato J., Jung Y.H., Kim Y.K., Nahm B.H., Jwa N.S., Tamogami S., Han O., Kohda K. (2007). Survey of differentially expressed proteins and genes in jasmonic acid treated rice seedling shoot and root at the proteomics and transcriptomics levels. J. Proteome Res..

[66] Walls R., Appel H., Cipollini M., Schultz J. (2005). Fertility, root reserves and the cost of inducible defenses in the perennial plant *Solanum carolinense*. J. Chem. Ecol..

[67] Skrzypek E., Miyamoto K., Saniewski M., Ueda J. (2005). Jasmonates are essential factors inducing gummosis in tulips: Mode of action of jasmonates focusing on sugar metabolism. J. Plant Physiol..

[68] Babst B.A., Ferrieri R.A., Gray D.W., Lerdau M., Schlyer D.J., Schueller M., Thorpe M.R., Orians C.M. (2005). Jasmonic acid induces rapid changes in carbon transport and partitioning in Populus. New Phytol..

[69] Hanik N., Gomez S., Best M., Schueller M., Orians C.M., Ferrieri R.A. (2010). Partitioning of new carbon as C-11 in *Nicotiana tabacum* reveals insight into methyl jasmonate induced changes in metabolism. J. Chem. Ecol..

[70] Tytgat T.O., Kjf V., Jansen J.J., Raaijmakers C.E., Bakxschotman T., Mcintyre L.M., Wh V.D.P., Biere A., van Dam N.M. (2013). Correction: Plants know where it hurts: Root and shoot jasmonic acid induction elicit differential responses in *Brassica oleracea*. PLoS ONE.

[71] van Dam N.M., Oomen W.A.T. (2008). Root and shoot jasmonic acid applications differentially affect leaf chemistry and herbivore growth. Plant Signal. Behav..

[72] Kim J., Quaghebeur H., Felton G.W. (2011). Reiterative and interruptive signaling in induced plant resistance to chewing insects. Phytochemistry.

[73] Pineda A., Dicke M., Pieterse C.M.J., Pozo M.J. (2013). Beneficial microbes in a changing environment: Are they always helping plants to deal with insects?. Funct. Ecol..

[74] de Vos M., Van Oosten V.R., Van Poecke R.M., Van Pelt J.A., Pozo M.J., Mueller M.J., Buchala A.J., Métraux J.P., Van Loon L.C., Dicke M. (2005). Signal signature and transcriptome changes of *Arabidopsis* during pathogen and insect attack. Mol. Plant Microbe Interact..

[75] Walling L.L. (2000). The myriad plant responses to herbivores. J. Plant Growth Regul..

[76] Herrera-Medina M.J., Tamayo M.I., Vierheilig H., Ocampo J.A., García-Garrido J.M. (2008). The jasmonic acid signalling pathway restricts the development of the arbuscular mycorrhizal association in tomato. J. Plant Growth Regul..

[77] Song Y.Y., Ye M., Li C.Y., Wang R.L., Wei X.C., Luo S.M., Zeng R.S. (2013). Priming of anti-herbivore defense in tomato by arbuscular mycorrhizal fungus and involvement of the jasmonate pathway. J. Chem. Ecol..

[78] Jacobs S., Zechmann B., Molitor A., Trujillo M., Petutschnig E., Likpa V., Kogel K.H., Schäfer P. (2011). Broad-spectrum suppression of innate immunity is required for colonization of Arabidopsis Roots by the fungus *Piriformospora indica*. Plant Physiol..

[79] Barazani O., Benderoth M.K., Kuhlemeier C., Baldwin I.T. (2005). *Piriformospora indica* and *Sebacina vermifera* increase growth performance at the expense of herbivore resistance in *Nicotiana attenuata*. Oecologia.

[80] Cosme M., Lu J., Erb M., Stout M.J., Franken P., Wurst S. (2016). A fungal endophyte helps plants to tolerate root herbivory through changes in gibberellin and jasmonate signaling. New Phytol..

[81] Porter J.K., Bacon C.W., Cutler H.G., Arrendale R.F., Robbins J.D. (1985). In vitro auxin production by *Balansia epichloë*. Phytochemistry.

[82] De Battista J.P.D., Bacon C.W., Severson R., Plattner R.D., Bouton J.H. (1990). Indole acetic acid production by the fungal endophyte of tall fescue. Agron. J..

[83] Yue Q., Miller C.J., White J.F., Richardson M.D. (2000). Isolation and characterization of fungal inhibitors from *Epichloë festucae*. J. Agric. Food Chem..

[84] Yang D.L., Yao J., Mei C.S., Tong X.H., Zeng L.J., Li Q., Xiao L.T., Sun T.P., Li J., Deng X.W. (2012). Plant hormone jasmonate prioritizes defense over growth by interfering with gibberellin signaling cascade. Proc. Natl. Acad. Sci. USA.

[85] Matschi S., Hake K., Herde M., Hause B., Romeis T. (2015). The calcium-dependent protein kinase CPK28 regulates development by inducing growth phase-specific, spatially restricted alterations in jasmonic acid levels independent of defense responses in Arabidopsis. Plant Cell.

[86] Heinrich M., Hettenhausen C., Lange T., Wünsche H., Fang J., Baldwin I.T., Wu J. (2013). High levels of jasmonic acid antagonize the biosynthesis of gibberellins and inhibit the growth of *Nicotiana attenuata* stems. Plant J..

[87] Ren C.G., Dai C.C. (2012). Jasmonic acid is involved in the signaling pathway for fungal endophyte-induced volatile oil accumulation of *Atractylodes lancea* plantlets. BMC Plant Biol..

[88] Latch G.C.M., Christensen M.J., Samuels G.J. (1984). Five endophytes of *Lolium* and *Festuca* in New Zealand. Mycotaxon.

[89] Kannadan S., Rudgers J.A. (2008). Endophyte symbiosis benefits a rare grass under low water availability. Funct. Ecol..

[90] Hoagland D.R., Arnon D.I. (1938). The water-culture method for growing plants without soil. Circ. Calif. Agric. Exp. Stat..

[91] Zhou Y., Li X., Gao Y., Liu H., Gao Y.B., van der Heijden M.G.A., Ren A.Z. (2018). Plant endophytes and arbuscular mycorrhizal fungi alter plant competition. Funct. Ecol..

[92] Buysse J., Merckx R. (1993). An improved colorimetric method to quantify sugar content of plant tissue. J. Exp. Bot..

[93] Yu L.M., Wang C.K., Wang X.C. (2011). Allocation of nonstructural carbohydrates for three temperate tree species in Northeast China. Chin. J. Plant Ecol..

[94] Thaler J.S., Stout M.J., Karban R., Duffey S.S. (1996). Exogenous jasmonates simulate insect wounding in tomato plants (*Lycopersicon esculentum*) in the laboratory and field. J. Chem. Ecol..

[95] Assis J.S., Maldonado R., Muñoz T., Escribano M.A.I., Merodio C. (2001). Effect of high carbon dioxide concentration on PAL activity and phenolic contents in ripening cherimoya fruit. Postharvest Biol. Technol..

